# STING activation in renal and prostatic inflammation: potential therapeutic targets and immune regulation

**DOI:** 10.3389/fimmu.2026.1743707

**Published:** 2026-04-14

**Authors:** Shilong Cao, Zhuoling Kong, Haoyuan Zheng, Peng Xin, Yutao Wang, Jianfeng Wang

**Affiliations:** 1 Department of Urology, The First Hospital of China Medical University, Shenyang, China; 2 Institute of Urology, China Medical University, Shenyang, China; 3 Department of Urology, Peking Union Medical College Hospital, Chinese Academy of Medical Sciences and Peking Union Medical College, Beijing, China

**Keywords:** immunotherapy, innate immunity, kidney disease, prostatic inflammation, renal inflammation, STING pathway

## Abstract

The cyclic GMPAMP synthase (cGAS) stimulator of interferon genes (STING) pathway is a central component of innate immunity that links cytosolic DNA sensing to type I interferon and NF-κB-driven inflammatory responses. Although transient STING stimulation facilitates antimicrobial defense, tissue remodeling, and tumor immunosurveillance, sustained or maladjusted signaling facilitates chronic sterile inflammation, fibrosis, immune impairment, and carcinogenesis. Mitochondrial injury, cellular senescence, infection-related stress, and DNA damage are caused by STING in epithelial-rich organs like the kidney and prostate, which can cause inflammatory diseases and context-dependent immunomodulation in cancer. This mini-review provides an integrated view of STING activation in renal and prostate tissues, elucidating common mechanistic activators, distinct pathological outcomes, and new translational possibilities. Most recent therapeutic strategies, such as STING agonists to promote antitumor immunity and STING inhibitors to reduce maladaptive inflammation. Further insight into cell-specific and disease-stage-dependent STING regulation will be critical for developing safe and effective interventions to achieve immune homeostasis in renal and prostate pathologies.

## 1 Introduction

Intrinsic immunity provides the first line of cellular defense against microbial attacks and pathological destruction. Some of its prerequisite sensors include the cGAS-STING pathway, which detects microbial or self-DNA in the cytosol (1, 2). The cGAS binding to DNA causes the production of cyclic GMP (cGAMP), which activates the endoplasmic reticulum adaptor protein STING (3). STING activation stimulates STING trafficking from the ER to the Golgi, recruiting TBK1 and IRF3, thereby triggering the production of type I interferons and NF-κB-regulated cytokines that mediate antiviral and antitumor immunity (4).

STING activation may trigger autoimmune and inflammatory diseases, including systemic lupus erythematosus, atherosclerosis, and neuroinflammation such as Alzheimer’s disease and Parkinson’s disease, which are challenging to treat (5, 6). STING leads to tissue destruction, inflammation, and fibrosis of the kidney and prostate. STING is induced by mitochondrial damage and self-DNA in renal tubular cells (7, 8). Conversely, STING signaling is triggered by infection, senescence, or tumor-derived DNA in prostate tissue, thereby influencing inflammatory and immune responses (9).

Recently single-cell and transcriptomic studies have shown that cell-specific STING is expressed in these organs, suggesting that it may be a potential cause of disease (10). In addition, pharmacological therapy for inflammatory kidney disease and prostate cancer, utilizing small-molecule agonists or inhibitors of STING modulation, is under development (11, 12). This study presents mechanistic, pathology-relevant, and novel STING-targeted therapeutic interventions designed to restore immune homeostasis and tissue integrity.

Importantly STING activation has been extensively investigated in kidney-related diseases, including renal inflammation and chronic kidney disease. However, the cGAS-STING pathway is rarely studied from the perspective of kidney-prostate crosstalk. Both organs are epithelial tissues and share susceptibility to DNA-stress induced pathological effects (13). In the kidney and prostate, multiple factors can activate the cGAS-STING axis, such as mitochondrial dysfunction, cellular senescence, infection-associated injury and cytosolic DNA accumulation. These activators further trigger type I interferon and NF-κB-mediated inflammatory programs. Such programs drive immune cell recruitment, cytokine amplification and tissue remodeling in both organs (14). STING signaling exerts context-dependent effects: transient activation supports antimicrobial defense and tumor immunosurveillance, while chronic or uncontrolled stimulation induces sterile inflammation, fibrosis, immune dysregulation and carcinogenesis (14, 15). This high context dependence poses a key translational question for clinical practice: when should STING be inhibited to reduce inflammatory tissue damage, and when can targeted STING activation be utilized to boost antitumor immunity? To address this question, this mini-review adopts a unified and comparative approach to analyze STING-mediated innate immune activation in renal and prostatic inflammation. We highlight common pathways, divergent disease responses, and opportunities for novel therapeutic development (16, 17).

## 2 Mechanistic overview of STING activation

**Figure 1:**
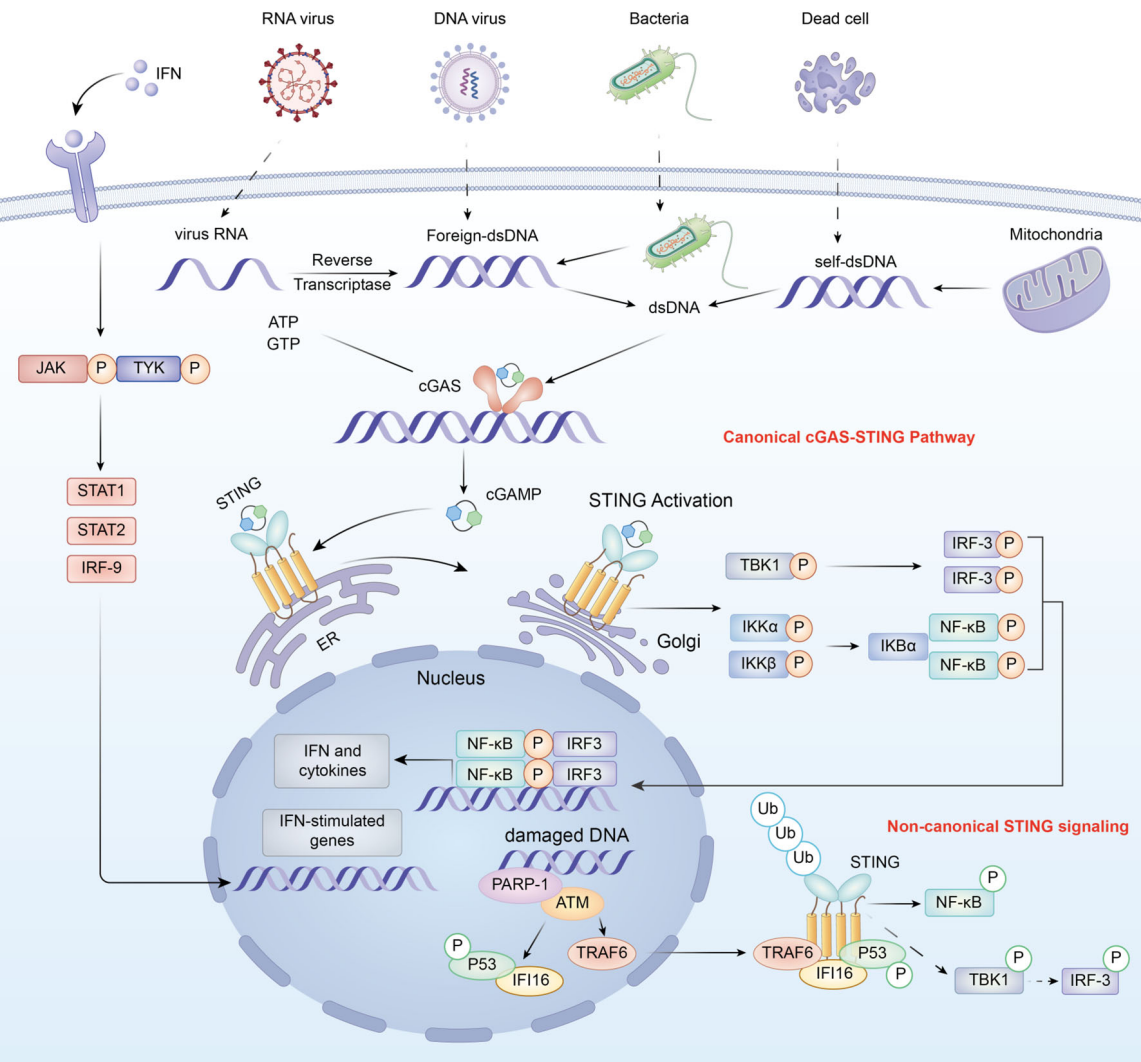
Schematic of the canonical cGAS-STING signaling pathway leading to type I interferon production. Cytosolic pathogenic DNA, mitochondria, or injured nuclei stimulate cGAS to produce cGAMP, which interacts with STING on the endoplasmic reticulum, leading to the activation of TBK1 and IRF3 and the production of type I interferons and pro-inflammatory cytokines. The image is used under a Creative Commons Attribution (CC BY) license from Shen, M., Jiang, X., & Peng, Q. (2025) (22).

Transmembrane protein 173 (*TMEM173*) is a protein in the carotid artery that enhances the innate immune system by triggering interferon gene packages via the integration of STING (18, 19). It has been discovered and contributes to understanding the mechanisms of the immune system’s response to nucleic acids that trigger type I interferon (IFN) responses. STING is primed to respond to cytosolic double-stranded DNA (dsDNA) under resting conditions, a response mediated by cGAS (20). *CGAS* binding to DNA leads to the production of 2-AMP-cGAMP, a cyclic dinucleotide (CDN) that stimulates STING through its ligand-binding receptor (21). **Figure 1** presents both the canonical and non-canonical cGAS-STING signaling pathways that involve the recognition of cytosolic DNA, STING activation, trafficking, and the downstream stimulation of type I interferons and cytokines regulated by NF-κB (22).

### 2.1 Activation and trafficking

STING also undergoes conformational changes and oligomerizes upon binding cGAMP, thereby facilitating intracellular delivery. STING enters the ER into ER-Golgi intermediate compartment (ERGIC) and Golgi apparatus via coat protein complex II (COPII) vesicles, which are regulated by Sec23/24 and TOLLIP proteins (23). The former occurs in the Golgi apparatus, where STING uses TANK-binding kinase 1 (TBK1), which phosphorylates STING and interferon regulatory factor 3 (IRF3) (24). Dimerization upon phosphorylation of IRF3 is followed by translocation to the nucleus and induction of transcription of interferon type I (IFNs) and interferon-stimulated genes (ISGs). NF-κB signaling enhances the production of inflammatory cytokines, such as TNF-α, IL-6, and CXCL10 (25).

### 2.2 Resolution and degradation

STING activation is also regulated to prevent overexuberant inflammatory responses. Signal transduction triggers the release of STING to lysosomes for degradation, a process that requires autophagy and the action of lysosomal enzymes (6). Dysfunction of this termination pathway extends STING activity and chronic inflammatory conditions that are typical of autoimmune diseases and non-infectious tissue injury, such as renal and prostate diseases (26). Post-translational modifications that affect STING stability and localization include ubiquitination, palmitoylation, and phosphorylation. In particular, deactivation occurs only when Cys88 and Cys91 of the Golgi apparatus are palmitoylated. Depending on the nature of the attachment and conditions, ubiquitination can result in the degradation or stabilization of the STING protein (27).

### 2.3 Crosstalk with cellular stress and metabolism

In addition to detecting pathogens, STING serves as a signal hub in response to other forms of stress. Renal tubular epithelial cell mitochondrial dysplasia leads to the release of mitochondrial DNA (mtDNA) into the cytosol, thereby activating the cGAS-STING pathway (28). Similarly, STING-based inflammation in diabetic and ischemic kidneys is facilitated by oxidative stress. STING can be activated by cellular senescence, radiation, or tumor-generated DNA in epithelial and stromal cells in prostate tissues, indicating that the DNA damage response is connected to inflammation and cancer (2, 29). STING signaling also mediates metabolic reprogramming in macrophages and dendritic cells, altering the immunologic microenvironment by regulating glycolysis and lipid metabolism (12, 30).

### 2.4 Context-dependent immune outcomes

Activation of STING is beneficial in acute infections and tumor immunosurveillance; however, prolonged activation can be detrimental to inflammation, leading to tissue damage. Therefore, STING is a double-edged sword that should be controlled with spatiotemporal precision to ensure accuracy (31). The basis of its therapeutic use in renal and prostate diseases is its dual role, which is both protective and pathogenic (32).

## 3 STING activation in renal inflammation

Kidneys are immunologically and metabolically active organs that are exposed to stressors, which activate the innate immune system (33). The relationship between mitochondrial dysfunction, cytosolic DNA accumulation, and sterile inflammation of renal tissues has recently been reported to be mediated by the cGAS-STING axis (34). Low STING expression is usually observed in tubular epithelial cells, glomerular podocytes, and resident macrophages (35). Nevertheless, leakage of mtDNA or damaged nuclear DNA into the cytosol occurs during stress or damage, thereby activating *cGAS*. This leads to the formation of cGAMP, which in turn activates STING. This cascade facilitates the production of IFN, the release of NF-κB-stimulated cytokines, and inflammation, as well as the remodeling of kidney tissue (36).

### 3.1 STING in acute kidney injury

Ischemia-reperfusion injury (IRI) leads to the release of cytosolic mtDNA, which triggers the STING pathway in tubular epithelial and dendritic cells. STING triggers TBK1-IRF3, leading to the production of interferon-2 (IFN-2) and chemokines, including CXCL10, which induces inflammatory responses in neutrophils and macrophages (37). Mice with STING deficiency showed less tubular necrosis, inflammatory infiltration, and renal dysfunction following IRI, implying that STING activation worsens acute renal injury (38). Similar results have also been reported by other researchers on cisplatin-induced nephrotoxicity, in which STING inhibition suppressed renal inflammation and apoptosis (7).

### 3.2 STING in autoimmune and chronic kidney diseases

Activation of the *cGAS*-STING pathway mediates the systemic lupus erythematosus (SLE) interferon signature in lupus nephritis (LN). The activation of the cGAS pathway is performed by autoantibody-DNA complexes and apoptotic debris (39). STING, TBK1, and IRF3 expression was associated with disease activity in murine models and human LN biopsies (40). STING inhibition lowers IFN-β expression and alleviates renal pathology in lupus-prone mice. High glucose levels in diabetic kidney disease (DKD) induce mitochondrial injury, which releases mtDNA and activates the STING pathway in proximal tubular cells (41). STING activation enhances the production of inflammatory cytokines, including IL-6, TNF-α, and TGF-β, which promote fibrosis. Renal fibrosis is reduced in diabetic mice treated with C-176 or H-151, STING inhibitors (42, 43).

### 3.3 Crosstalk with immune cells

The STING pathway in macrophages and dendritic cells is coordinated to produce a pro-inflammatory renal microenvironment (44). Resident macrophages exposed to mtDNA or oxidized DNA exhibit an M1-like phenotype and release TNF-α and IL-1β, which further enhances tubular cell damage (7). Activation of STING in dendritic cells enhances antigen presentation and T cell priming, thereby sustaining autoimmunity in lupus and chronic nephritis. It has been suggested that STING regulates macrophage metabolism by promoting glycolysis and reactive oxygen species production, while also enhancing inflammatory feedback responses (45).

### 3.4 Regulatory and protective mechanisms

Despite the pathogenic potential of STING activation, transient activation is essential for tissue repair. In particular, controlled interferon signaling can help eliminate necrotic debris and promote tubule regeneration in the kidneys. Therefore, therapeutic approaches that adjust rather than block STING should be considered to achieve a balance between immune defense and tissue conservation. The primary challenge in identifying STING activity thresholds is defining disease-specific thresholds that distinguish protective from detrimental responses. **Table 1** summarizes the experimental and clinical models linking STING activation to renal inflammation.

**Table 1:** Experimental and clinical evidence linking STING activation to renal inflammation.

Model/condition	Key cell types involved	Mechanism of STING activation	Major pathologic outcomes	Therapeutic/genetic intervention	Status/notes
IRI, mouse	Tubular epithelial cells, dendritic cells	Mitochondrial DNA (mtDNA) leakage → cGAS activation → STING-TBK1-IRF3 pathway	↑ IFN-β, CXCL10, neutrophil/macrophage infiltration, tubular necrosis	STING knockout or H-151 treatment reduces injury	The canonical model of sterile activation demonstrates mtDNA-triggered inflammation (46)
Cisplatin-Induced Nephrotoxicity	Tubular epithelial cells	DNA damage and mitochondrial stress → cytosolic DNA accumulation	↑ TNF-α, IL-6, apoptosis, renal dysfunction	C-176 or C-178 lowers cytokine release and apoptosis	Supports the role of STING in drug-induced AKI and nephrotoxicity (36)
Lupus Nephritis (MRL/lpr, NZB/W F1 mice; human LN biopsies)	Podo-cytes, macrophages, dendritic cells	DNA immune complexes and apoptotic debris → cGAS-STING activation	↑ Type I IFN signature, glomerular inflammation, and immune complex deposition	Genetic or pharmacologic STING blockade ameliorates disease	Correlates with IFN score and disease severity in human LN (39)
DKD (STZ or db/db mice)	Proximal tubular cells, mesangial cells	mtDNA release and oxidative stress → cGAS-STING activation	↑ NF-κB activation, TGF-β1, collagen I, renal fibrosis	H-151 or C-176 suppresses fibrosis and albuminuria	Links metabolic stress and mitochondrial injury to innate immune activation (47)
Unilateral Ureteral Obstruction (UUO)	Macrophages, fibroblasts	Damage-associated molecular patterns (DAMP)-induced cytosolic DNA sensing via cGAS-STING	↑ IL-6, MCP-1, and extracellular matrix deposition	STING deletion limits fibrosis and cytokine expression	Demonstrates fibrotic potential of sustained STING signaling (48)
Adenine-Induced Chronic Kidney Disease	Tubular epithelial cells, macrophages	Crystalline injury and mitochondrial rupture → cytosolic DNA accumulation	↑ IFN-β, IL-1β, fibrotic remodeling, and tubular atrophy	STING-deficient mice protected from fibrosis	Highlights the role in sterile chronic kidney injury and crystal nephropathy (7)
Sepsis-Associated AKI (Cecal Ligation and Puncture model)	Endothelial cells, macrophages	Bacterial DNA and cGAMP accumulation activate STING	↑ IFN-β, vascular leakage, tubular apoptosis, inflammation	STING inhibitor lowers mortality and cytokine storm	Represents an infectious activation context distinct from sterile injury (31)
Aging-Related Renal Inflammation	Tubular epithelial and interstitial cells	Cytoplasmic chromatin fragments in senescent cells → cGAS-STING activation	↑ SASP cytokines (IL-6, IL-8), interstitial fibrosis	Genetic deletion of STING delays senescence-driven inflammation	Mirrors findings in aging prostate; links DNA damage and inflammation (49)
Human CKD Biopsies (various etiologies)	Tubular epithelium, infiltrating macrophages	Elevated *TMEM173* (STING) transcripts, mitochondrial stress markers	Correlates with interstitial inflammation and fibrosis	Observational (no direct intervention)	Suggests translational relevance of STING pathway activation in human CKD (50)

### 3.5 STING signaling in renal cancer and tumor-associated inflammation

Although STING activation has been characterized primarily in sterile and autoimmune kidney disease, there is now evidence of the involvement of the cGAS-STING pathway in renal malignancy, namely renal cell carcinoma (RCC). STING signaling is also highly context-specific in RCC: dendritic cell and antigen-presenting cell activation can promote type I interferon generation and T-cell priming, thereby supporting antitumor immune surveillance and potentially increasing immunotherapy responsiveness (14, 17). In contrast, chronic STING activity in the tumor microenvironment can reinforce chronic inflammation, immune exhaustion, and tumor-stimulating cytokine programs, thereby evading immunity and promoting further progression (16). Transcriptomic analysis also indicates changes in STING pathway expression in RCC, which are associated with immune infiltration and patient prognosis (17). In line with this, pharmacologic control of STING has been considered in RCC, either by agonistic stimulation to augment antitumor immunity or by inhibition to suppress tumor remodeling in inflammatory conditions (13, 17).

## 4 STING activation in prostatic inflammation and cancer

The prostate is an active immune and endocrine gland, and its cells are embedded in a microenvironment sensitive to infection and tissue damage (5). New evidence indicates that unintended activation of the STING pathway contributes to the pathogenesis of prostate inflammation and regulates the immune environment in both malignant and benign phenotypes (51). As in the kidney, cytosolic DNA sensing via the cGAS-STING pathway in the prostate links cell injury, senescence, and microbial infection, thereby initiating innate immune activation and cytokine signaling (12).

### 4.1 Distinguishing benign prostatic inflammation from cancer-associated inflammation

Inflammation of the prostate. Benign prostatic inflammation, such as chronic bacterial prostatitis, chronic pelvic pain syndrome, and asymptomatic inflammatory prostatitis, is characterized by immune-cell infiltration and epithelial-to-stromal activation without malignancy and may involve sterile activation of the cGAS-STING pathway by mitochondrial DNA release or hormonal stressors (52–54). However, prostate cancer-related inflammation is a manifestation of tumor-immune co-evolution, with STING signaling being highly context-dependent: it can either support antitumor immunity via type I interferon-mediated dendritic cell activation or, in dysregulation, facilitate tumor-associated inflammation and immunosuppressive restructuring of the microenvironment (55–58).

### 4.2 Infectious and chronic prostatitis STING

Prostatitis, particularly chronic pelvic pain syndrome and chronic bacterial prostatitis, is associated with inflammation and immune cell involvement (59). Bacterial infections release pathogen-associated molecular patterns and self-DNA, which trigger the cGAS-STING pathway. STING activation leads to the production of interferon-α, TNF-α, and IL-6 in murine models of *E. coli*-induced prostatitis, which, in turn, recruits immune cells. STING-deficient mice showed reduced inflammation, suggesting that STING is involved in the inflammatory response. The STING pathway is activated by senescent cells in chronic non-bacterial prostatitis. Cytoplasmic chromatin buildup within senescent prostate cells triggers the activation of NF-κB through the cGAS-STING pathway, leading to the senescence-associated secretory phenotype (SASP) (60). This leads to inflammation, and cytokines recruit neutrophils and macrophages, which then mediate tissue remodeling and cause pain (61).

### 4.3 Role of STING in the microenvironment of PC tumors

The STING pathway is context-sensitive in PC. STING-induced dendritic cell activation is associated with enhanced antitumor immunity, facilitating IFN production and enabling T cell infiltration (62). However, in murine prostate tumor models, chronic STING activation by cyclic dinucleotides (CDNs) may paradoxically promote immunosuppressive checkpoint pathways within the tumor microenvironment, thereby limiting effective antitumor immunity (63). Nevertheless, the potential of long-term STING signaling lies in stimulating tumor development by inducing tumor growth through inflammation-mediated EMT, angiogenesis, and the recruitment of MDSC, which facilitates immune evasion (64). In other PC cells, dysfunctional IFN signaling has been observed despite STING expression. DNA-damaging therapies can release cytosolic DNA, which activates the STING pathway in cancer cells and modulates the antitumor response (65).

### 4.4 Crosstalk between STING and androgen axis

The prostate’s dependence on androgen signaling provides additional regulatory factors. Androgen receptors (ARs) also contribute to innate immune responses, and evidence suggests that AR signaling can suppress STING signaling (66). Androgens in androgen-sensitive PC cells suppress STING and cGAS expression, thereby inhibiting immune activation in the tumor microenvironment (TME). In contrast, AR blockade or androgen deprivation augments STING signaling, suggesting a mechanism by which androgen-directed therapy can be combined with STING agonists to restore antitumor immunity in patients with PC (67).

### 4.5 Implications for translation

**Figure 2:**
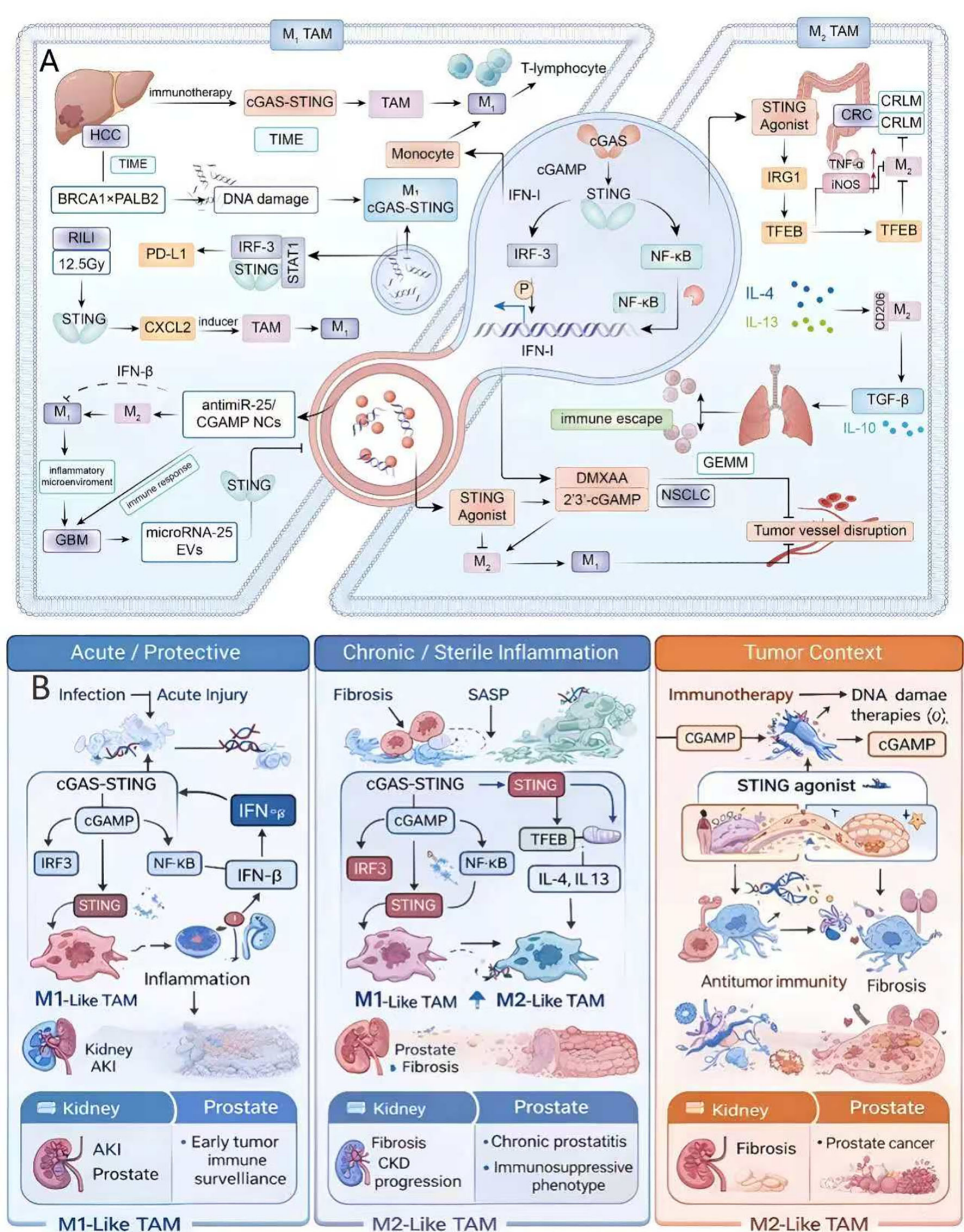
**(A)** Dual role of STING signaling in inflammation and tumor immunity. STING activation in M1 macrophages enhances type I interferon and antitumor responses, whereas chronic activation in M2 macrophages induces IL-4/IL-13-mediated immunosuppression, fibrosis, and tumor progression. Reprinted from Wang, Q., Yu, Y., & Zhuang, J. (2025) under the CC Licensing (69), **(B)** Context-dependent outcomes of STING activation across kidney and prostate inflammation and cancer.

Complicated STING signaling is a current approach for targeting prostate diseases. STING agonists have the potential to be used in tumor immunotherapy to reprogram the immune microenvironment, thereby enhancing the effectiveness of checkpoint inhibitors. In contrast, STING inhibitors may suppress chronic inflammation in prostatitis, thereby alleviating pain and tissue damage (68). Detailed insights into the temporal and spatial aspects of STING receptor activation, as well as the differences between acute, chronic, epithelial, and immune cell-mediated reactions, are vital for the development of targeted treatments. **Figure 2** illustrates the context-dependent functions of STING signaling in tumor immunity and macrophage polarization, where M1 activation promotes interferon production and antitumor eradication (69). In contrast, M2 polarization leads to immunosuppression and fibrotic remodeling. **Figure 2B** extends this framework by integrating kidney-prostate disease contexts and highlighting how epithelial- and immune cell-driven STING signaling diverges across acute injury, chronic sterile inflammation, prostatitis, and prostate cancer, with corresponding therapeutic implications (STING inhibition vs agonism).

## 5 Therapeutic targeting of the STING pathway

The cGAS-STING axis plays a dual role: it serves as a protective host defense mechanism and becomes pathogenic when dysregulated (70). This has made therapeutic regulation a significant target in studies of immune oncology and inflammatory diseases. Pharmacological targeting of STING can be pursued in two opposing directions: agonism to promote antitumor and antiviral immune responses, and inhibition to repress aberrant inflammation in autoimmune or sterile inflammatory diseases, including renal and prostate diseases (71, 72). Accordingly, pharmacological modulation has expanded in two complementary directions: agonism to enhance interferon-driven antitumor immunity and inhibition to suppress maladaptive sterile inflammation. Early clinical STING agonists have largely been cyclic dinucleotide (CDN)–based agents administered intratumorally to reduce systemic toxicity, including MIW815/ADU-S100 and MK-1454 (ulevostinag), and have often been evaluated in combination with immune checkpoint blockade (73). Additional agonists, such as E7766, have demonstrated engagement of the pharmacodynamic pathway in first-in-human studies (74). At the same time, next-generation strategies include non-nucleotide small-molecule agonists (e.g., diABZI scaffolds) and engineered delivery platforms designed to improve tissue targeting (2, 9). Conversely, anti-inflammatory approaches focus on limiting STING activation or trafficking: covalent inhibitors that disrupt STING palmitoylation (C-176/C-178) provide early proof of concept (10), and newer non-covalent antagonists, such as SN-011, reduce cytokine production in preclinical models (11). Collectively, these advances underscore the need for tissue- and disease-stage–specific tuning of STING activity, enhancing it in prostate cancer immunotherapy while dampening it in sterile renal inflammation, rather than uniform pathway activation or blockade (14, 17).

### 5.1 STING agonists

STING agonists stimulate interferon synthesis and increase dendritic cell maturation and T cell activation. Activators have been identified as natural CDNs, including 2′3′-cGAMP; however, they are unstable in their native form. ADU-S100, MK-1454, and diABZI are more potent synthetic derivatives. Agonists have been tested in solid tumors, such as PC, where they disrupt the TME and augment checkpoint blockade therapy (75). Intratumoral CDN injection inhibited PC growth and augmented the antitumor effects of PD-1 and CTLA-4 inhibition in PC models. Combination therapies can also be used, as radiotherapy can supplement the action of STING agonists, thereby increasing cytosolic DNA (76).

STING agonists include endogenous cyclic dinucleotides such as 2′3′-cGAMP, although poor stability and delivery constraints limit their clinical utility. More potent synthetic agonists, including ADU-S100, MK-1454, and non-nucleotide diABZI derivatives, have therefore been developed and evaluated in early-phase studies of solid tumors, including prostate cancer. In preclinical models, intratumoral STING activation can remodel the tumor microenvironment and enhance responses to immune checkpoint blockade. Combining radiotherapy with agonist strategies may further enhance agonist efficacy by increasing cytosolic DNA release and amplifying STING-dependent interferon signaling (75, 77).

### 5.2 STING inhibitors

STING inhibitors are currently under development to inhibit cytosolic DNA-driven maladaptive inflammation that is a contributor to sterile tissue injury, autoimmune pathology, and fibrotic remodeling in renal and benign prostate disease. Unlike in cancer models, where a restrained immune response can be advantageous, chronic kidney inflammation and prostatitis typically exhibit sustained STING signaling; thus, blocking these pathways is an effective treatment strategy. Nonetheless, antimicrobial defense and immune surveillance can be impaired by long-term blockade, so it should be carefully balanced.

C-176/C-178 (murine) and the human-selective antagonist H-151 are several small-molecule inhibitors that suppress STING signaling by inhibiting essential activation events, including palmitoylation, thereby suppressing downstream TBK1-IRF3 and NF-κB inflammatory outputs (63). STING inhibition in experimental models of lupus nephritis and diabetic kidney disease has been shown to reduce interferon signatures, inflammatory cytokine production, and fibrosis-associated remodeling (64). Other modalities- targeting upstream cGAS activity or improving pathway termination were also investigated as an attempt to refine cytosolic DNA -sensing inflammation (78).

Overall, these findings support STING inhibition as a rational anti-inflammatory strategy, but emphasize the need for biomarker-guided patient selection and careful monitoring of infection risk.

### 5.3 Translational perspective

To maximize therapeutic approaches, a critical assessment of the disease is required. STING inhibition is likely beneficial for renal and prostate inflammation; however, STING agonists are used in PC treatment (79). Essential aspects to consider include tissue-specific STING isoform expression, downstream signaling dynamics, potential cross-regulation, and hormonal and metabolic factors (80). In the future, advances in organ-targeted delivery systems and STING activation biomarkers are expected to improve the clinical translation and safety of STING-based therapies. **Table 2** presents the representative STING-targeted pharmacologic compounds (agonists and inhibitors) along with their current preclinical or clinical development status.

**Table 2:** Representative STING modulators and their preclinical and clinical status.

Compound/class	Mechanism of action	Therapeutic aim	Disease/experimental model	Stage/status	Key findings/notes
2′3′-cGAMP (natural CDN)	Endogenous cyclic dinucleotide binding to STING	STING agonist; activates TBK1–IRF3	Viral infection, cancer immunotherapy	Preclinical	First discovered natural ligand; limited by poor stability and systemic delivery (81)
ADU-S100 (MIW815)	Synthetic CDN agonist (phosphorothioate-stabilized)	Enhances antitumor immunity	Solid tumors (melanoma, head/neck, prostate)	Phase I/II	Promotes IFN-β and T-cell infiltration; synergizes with checkpoint inhibitors (82)
MK-1454	CDN analog with improved pharmacokinetics	STING activation for tumor immunity	Solid tumors, lymphoma	Phase I	Safe in local injection; potentiates PD-1 blockade (83)
diABZI-1/-2	Non-nucleotide small-molecule agonists	Systemic STING activation	Murine tumor and viral infection models	Preclinical/early clinical	High potency and bioavailability; excessive activation can cause cytokine toxicity (84)
E7766	Macrocyclic CDN agonist	Immunostimulant	Solid and hematologic malignancies	Phase I (NCT04144140)	Improved tumor penetration; induces durable IFN signaling (85)
C-176/C-178	Covalent inhibitors (murine) blocking STING palmitoylation	Anti-inflammatory; STING inhibition	Lupus nephritis, DKD	Preclinical	Reduce renal IFN-β, IL-6, and fibrosis; validate antifibrotic potential (7)
H-151	Human-selective covalent inhibitor	STING inactivation via palmitoylation blockade	Autoimmune and renal inflammation	Preclinical	Decreases IFN-β, TNF-α, IL-6; effective in humanized models (86)
SN-011	Non-covalent allosteric inhibitor	Dampens chronic STING activation	Monogenic interferonopathies	Preclinical	High selectivity; oral bioavailability; favorable safety profile (87)
Astin C	Natural cyclopeptide STING antagonist	Anti-inflammatory	Autoimmune and renal injury models	Preclinical	Derived from *Aster tataricus*; inhibits STING-dependent cytokine release (77)

## 6 Conclusion

In conclusion, the cGAS-STING pathway is a central regulator of innate immunity and inflammation in both renal and prostatic disease. Transient, tightly controlled STING activation can support antimicrobial defense, tissue repair, and tumor immunosurveillance. In contrast, persistent or dysregulated signaling promotes chronic sterile inflammation, cytokine amplification, fibrosis, immune dysregulation, and organ dysfunction. In the kidney, STING-mediated interferon and NF-κB-driven cytokine programs exacerbate injury in ischemic, autoimmune, and metabolic settings, whereas in the prostate, STING signaling plays a dual role, contributing to benign inflammatory conditions such as prostatitis and shaping antitumor immunity within the cancer microenvironment. This context dependence underscores the need for precision therapeutic modulation: STING inhibitors may hold promise in sterile renal and benign prostatic inflammation, whereas controlled STING agonism may enhance prostate cancer immunotherapy. Future studies defining cell-type–specific activation thresholds, robust biomarkers, and organ-targeted delivery strategies will be essential for translating STING-based interventions into safe and effective clinical therapies that restore immune homeostasis without compromising host defense.

Future clinical translation of cGAS-STING modulation in renal and prostatic disease will require precision, context-dependent pathway tuning rather than uniform activation or inhibition. Key priorities include the development of effective biomarkers to distinguish between protective and pathogenic STING signaling, enabling patient stratification and pharmacodynamic monitoring, which warrants inclusion. Simultaneously, improved delivery methods are required because systemic activation of STING can lead to inflammatory toxicity, and long-term inhibition of the system can impair antimicrobial defense; therefore, organ-targeted formulations and next-generation modulators could help widen therapeutic windows (16, 17). Lastly, clinical integration will likely require indication-specific combination strategies, including controlled STING agonism with checkpoint blockade in prostate cancer, compared with STING inhibition in sterile renal inflammation (14, 17). All these developments will be essential for converting STING-based interventions into safe and effective therapies in renal and prostate disease settings.
